# ﻿Five new and noteworthy species of Epidendroideae (Orchidaceae) from southwestern China based on morphological and phylogenetic evidence

**DOI:** 10.3897/phytokeys.235.111230

**Published:** 2023-11-20

**Authors:** Ji-Dong Ya, Wan-Ting Wang, Yun-Long Liu, Hong Jiang, Zhou-Dong Han, Ting Zhang, Hua Huang, Jie Cai, De-Zhu Li

**Affiliations:** 1 Germplasm Bank of Wild Species, Kunming Institute of Botany, Chinese Academy of Sciences, Lanhei Road 132, Heilongtan, Kunming, Yunnan 650201, China; 2 Academy of Biodiversity, Southwest Forestry University, Kunming, Yunnan 650224, China; 3 University of Chinese Academy of Sciences, Beijing 100049, China; 4 Yunnan Laboratory for Conservation of Rare, Endangered & Endemic Forest Plants, Public Key Labotatory of the National Forestry and Grassland Administration, Yunnan Academy of Forestry and Grassland, Kunming, Yunnan 650201, China; 5 CAS Key Laboratory for Plant Biodiversity and Biogeography of East Asia, Kunming Institute of Botany, Chinese Academy of Sciences, Kunming, Yunnan 650201, China; 6 Lijiang Alpine Botanical Garden, Kunming Institute of Botany, Chinese Academy of Sciences, Lijiang, Yunnan 674100, China; 7 Lijiang Forest Biodiversity National Observation and Research Station, Kunming Institute of Botany, Chinese Academy of Sciences, Lijiang, Yunnan 674100, China

**Keywords:** *Neottia*, *Gastrochilus*, *Papilionanthe*, Plastid phylogenomics, Taxonomy, Tibet, Yunnan

## Abstract

Five new orchid species from southwestern China’s Yunnan Province and the Tibetan Autonomous Region, *Neottialihengiae*, *Neottiachawalongensis*, *Papilionanthemotuoensis*, *Gastrochiluslihengiae*, and *Gastrochilusbernhardtianus*, are described and illustrated. To confirm their identities, and to resolve phylogenetic relationships, we sequenced the complete plastomes of these taxa with their congeneric species, adding new plastomes of three *Neottia* species, two *Papilionanthe* species and nine *Gastrochilus* species. Combined with published plastid sequences, our well-resolved phylogeny supported the alliance of *N.lihengiae* with the the *N.grandiflora* + *N.pinetorum* clade. *Neottiachawalongensis* is now sister to *N.alternifolia*, while *P.motuoensis* is closely related to *P.subulata* + *P.teres*. Conversely, phylogenetic analyses based on complete plastomes and plastid sequences showed inconsistent relationships among taxa in the genus *Gastrochilus*, but the two new species, *G.lihengiae* and *G.bernhardtianus* were supported by all datasets.

## ﻿Introduction

The Himalaya and Hengduan Mountains of southwestern China are iconic biodiversity hotspots of global significance (Liu et al. 2022). However, the species diversity of this region remains inadequately understood. In recent years, a considerable number of new plant species were described from these regions (Cai et al. 2019). Taking the Orchidaceae as an example, early floristic accounts of Tibet (Xizang) in the “Flora Xizangica” included only 64 genera totaling 191 species (Wu 1987). In contrast, a number of new species and new geographic records were reported over the last three decades (Wang et al. 2018; Liu et al. 2020a; Li et al. 2022, 2023), increasing the number of orchids in Tibet to 110 genera with 491 species in the latest checklist (Wang et al. 2023). Long-term and in-depth field investigations are still required to meet the urgent challenge of conserving rare species in these mountainous regions as their habitats undergo rapid changes.

The Orchidaceae is one of the largest families of angiosperms in the world, with approximately 190 genera and 1600 species in China (Jin et al. 2019). The Hengduan Mountains and the Himalaya, particularly counties of Gongshan, Shangri-La, and Lijiang (Yunnan) and Motuo (Medog) in Tibet (Xizang) are particularly rich in orchids (Zhang et al. 2015).

The genus *Neottia* Guett. a member of the Neottieae, was first established in 1754 consisting of a few, small, mycoheterotrophic orchids (Chen 1999). Recent molecular studies indicate that the autotrophic genus *Listera* Brown should be submerged within *Neottia* s.l. The monophyletic tribe Neottieae becomes the sister to the majority of the remaining members of the largest subfamily, Epidendroideae in the Orchidaceae (Pridgeon et al. 2005; Chen et al. 2009; Chase et al. 2015; Zhou and Jin 2018). There are 73 accepted species in *Neottia* s.l. distributed widely across the temperate and subarctic regions of the Northern Hemisphere, but the genus extends to northwest Africa, with a few species native to alpine regions in subtropical eastern Asia (Pridgeon et al. 2005; Chen et al. 2009; POWO 2023). Morphologically, *Neottia* is readily distinguished from other terrestrial epidendendroids by its two opposite to nearly opposite leaves (when present) or it is leafless. Each resupinate flower contains a curved column containing two sectile but naked pollinia (indicative of basal epidendroids).

The small genus *Papilionanthe* Schltr., a member of Vandeae (subtribe Aeridinae), was first described by Schlechter in 1915 based on *Vandateres* (Roxb.) Lindl. published previously in genus *Vanda* R.Br. The genus *Papilionanthes.s.* is distinguished from other genera in subtribe Aeridinae by multiple characters. It has fleshy and terete leaves, and a short inflorescence arising from a node opposite the leaf. The trilobate labellum is spurred. Its mid-lobe is often dilated and 2- or 3-lobed at its apex. The subterete and short column has a short foot. Pollinia are attached to a broadly triangular or subquadrate stipe which, in turn, is attached to a large and cellular viscidium (Chen et al. 2009; Pridgeon et al. 2014). Ten *Papilionanthe* species are recognized currently distributed from India eastward to south-central China and Malesia (POWO 2023).

The genus *Gastrochilus* D. Don is also a member of the subtribe Aeridinae and was established in 1825. It is characterized by monopodial growth, erect or pendulous stems and short axillary inflorescences. The labellum has a saccate hypochile. Two porate and globose pollinia are borne on a slender stipe (Chen et al. 2009; Pridgeon et al. 2014; Liu et al. 2019). Recent molecular studies of *Gastrochilus* show that traditional classification based on morphological characters is well supported (Liu et al. 2019). The genus consists of ca. 70 species, distributed through subtropical Asia, from Sri Lanka and India into the Himalaya, eastwards to southern China, southern Japan and southwards to the Philippines and Indonesian archipelago. Appoximately 40 species are found in China (Chen et al. 2009; Liu et al. 2019; POWO 2023).

We collected specimens of five previously unidentified species during our field surveys in Yunnan and Tibet from 2016–2023. Following a review of the literature (see Pearce and Cribb 2002; Chen et al. 2009; Raskoti 2009; Jin and Pang 2016; Averyanov et al. 2018; Wu et al. 2019; Mu et al. 2020; Liao et al. 2022), morphological studies of herbarium specimens and plastid phylogenomic analyses, we concluded that these specimens are new to the genera *Neottia* (Orchidaceae: Epidendroideae, Neottieae), *Papilionanthe* and *Gastrochilus* (Orchidaceae: Epidendroideae, Vandeae, Aeridinae) respectively. These species are analyzed and described below.

## ﻿Materials and methods

### ﻿Morphological studies

Living plants and herbarium specimens were collected in the field in the Hengduan Mountains of northwestern Yunnan and the Himalaya of southeastern Tibet. Morphological characters and measurements of the specimens described here were based on at least 5 living specimens first observed in the field then cultivated plants in the greenhouse. Voucher specimens are deposited in the Herbarium of the Kunming Institute of Botany, Chinese Academy of Sciences (**KUN**) and the Herbarium of the Yunnan Academy of Forestry and Grassland (**YAF**).

### ﻿Taxon sampling, DNA extraction, sequencing, assembling and annotation

To clarify the phylogenetic relationships of five potentially new species with closely related species, we sampled and sequenced plastomes of 17 accessions representing three *Neottia* species, two *Papilionanthe* species and nine *Gastrochilus* species. Including those retrieved from the National Centre for Biotechnology Information (NCBI) database, our dataset comprises 412 plastid genes of a total of 83 accessions.

Total genomic DNA was extracted from silica-dried tissue using the Plant Genomic DNA Kit (Tiangen Biotech, Beijing, China). Libraries for pair-end 150 bp sequencing with 200–400 bp insert size were conducted on a BGISEQ-T7 platform at BGI Shenzhen (China) for genome skimming, producing approximately 2Gbp high-quality reads per sample. The plastomes of *Neottiaovata* (L.) Hartm. (NC_030712) and *Gastrochilusformosanus* (Hayata) Hayata (MN124435) were used as references for the assembling of the clean reads (Feng et al. 2016; Liu et al. 2020b). Complete plastomes and the nuclear internal transcribed spacer (ITS) assembly were conducted using the Getorganelle toolkit (Jin et al. 2020). The parameters used were R = 15, k = 21,45,65,85,105,127, F = embplant_pt; R = 7, k = 21,45,65,85,105,127, F = embplant_nr, respectively. Assembled plastid genomes were annotated by PGA GENEIOUS R9.0.2 (Biomatters Ltd. Auckland, New Zealand) using the plastome of *Neottiaovata*, *Holcoglossumamesianum* (Rchb. F.) Christenson (NC_041511.1) and *Gastrochilusformosanus* (Li et al. 2019).

### ﻿Phylogenetic analysis

For *Neottia*, a total of 22 taxa were included in the analysis of the data set comprising two plastid DNA (*mat*K, *rbc*L) and nuclear ribosomal (nr) ITS sequences, *Cephalantheralongifolia* (L.) Fritsch was used as the outgroup based on Zhou and Jin (2018). For *Papilionanthe*, phylogenomic analysis was implemented based on nrITS and six plastid markers (*mat*K, *trn*L-*trn*F, *psb*A-*trn*H, *atp*I-*atp*H, *trn*S-*trnf*M and *rbc*L) from 6 *Papilionanthe* species, including two newly sequenced species, and a total of 12 accessions representing four genera. They were all analyzed with *Ascocentrumampullaceum* (Roxb.) Schltr. as the outgroup (Zhang et al. 2013). For *Gastrochilus*, eight publicly available plastome sequences of *Gastrochilus* species were obtained from GenBank (Suppl. material 1) along with nine newly sequenced *Gastrochilus* species (n = 12 plants). Therefore, a total of 17 *Gastrochilus* plastomes were included in this study with *Pomatocalpaspicatum* Breda as the outgroup (Liu et al. 2019). DNA sequences obtained from nrITS and chloroplast *mat*K, *trn*L-*trn*F, *psb*A-*trn*H, *psb*M-*trn*D were combined as a data matrix. Voucher information and GenBank accession numbers are provided in Suppl. materials 1–4. The plastid genes were aligned individually using MAFFT v7.308 (Katoh and Standley 2013), and the alignment of ITS and plastid genes are available in ScienceDB, after which alignment columns used Gblocks 0.91(Castresana 2000). Before phylogenetic analysis, the best-fit Akaike Information Criterion (AIC) model was selected in JModelTest v2.1.10 (Darriba et al. 2012). Phylogenetic trees were constructed by maximum likelihood (ML) in RaxML v8.2.11 (Stamatakis 2014) with 1,000 bootstrap replicates and Bayesian inference (BI) methods in Mrbayes v3.2.6 (Ronquist et al. 2012). For Bayesian inference, two separate Markov chain Monte Carlo (MCMC) chains were run for 200,000 generations with mixed nucleotide substitution models and 25% (50,000) of the trees were deleted as burn-in, and the results of two independently run computations were merged to produce a Bayesian consistent tree and a posterior probability value (PP) for each branch.

## ﻿Results and discussion

### ﻿Characteristics of the plastomes

All newly sequenced plastomes were assembled completely and can be accessed from GenBank (Table 1). Their genome features were found to be nearly identical, and gene content is conserved with an identical set of 68 annotated unique protein-coding genes (except for *Neottia*) and 29–30 tRNA genes and 4 rRNA genes. While, all three newly sequenced *Neottia* plastome sizes ranged from 155,447–156,082 bp, their genomes were composed of an LSC region (84,270–84,930 bp), SSC region (17,875–18,113 bp) and two IR copies (26,367–26,682 bp), with 74–80 unique genes. Their overall G/C content was almost identical (37.5–37.6%). The total plastome lengths of *Papilionantheteres* (Roxb.) Schltr. and the putative new species, *P.motuoensis* ranged from 147,829–148,619 bp. Among all *Gastrochilus* plastomes, plastome sizes ranged from 146,615 to 148,552 bp. The genomes were composed of a large single repeat region (LSC) (84,710–85,682 bp), a small single repeat region (SSC), region (10,357–11,173 bp) and two inverted repeat (IR) copies (25,767–26,007 bp). Their overall G/C content was nearly identical (36.6–36.8%).

**Table 1.** Summary of plastomic data and nrITS sequences for *Neottia*, *Papilionanthe* and *Gastrochilus* species.

Species	GenBank accession number	Raw data	Genome size (bp)	LSC	SSC	IR	Number of unique protein coding genes	Number of tRNAs	Number of rRNA	ITS GenBank accession number	Sequence length [bp]
*Neottiachawalongensis*	OR786306	1.40/1.38G	155447	84581	18113	26367	119	30	4	OR073413	625
*N.lihengiae*	OR002177	4.71/4.70G	155600	84270	17969	26682	114	30	4	OR073414	623
*N.* sp.	OR002178	4.05/3.81G	156082	84930	17875	26639	109	30	4		623
*Papilionanthemotuoensis*	OR772949	1.64/1.58G	148,619	84,574	12,055	25,945	107	30	4	OR073415	668
*P.teres*	OR772950	1.00/1.02G	147,829	85,680	11,445	25,352	101	29	4	OQ991258	662
*Gastrochilusbernhardtianus*	OR772951	1.17/1.10G	147,078	84,845	10,357	25,938	101	29	4	OR073405	398
*G.bernhardtianus*	OR002167	1.70/1,72G	146,615	84,710	10,371	25,767	101	29	4	OR073404	654
*G.fargesii*	OR002175	1.55/1.54G	148,552	85,682	11,132	25,951	110	30	4		656
*G.distichus*	OR002170	873/893MB	147,834	85,063	11,113	25,829	101	29	4	OR073407	409
*G.distichus*	OR002171	975/914MB	147,826	85,010	11,112	25,852	101	29	4	OR073406	654
*G.gongshanensis*	OR002173	1.46/1.48G	147,728	84,936	11,032	25,880	101	29	4	OR073411	410
*G.gongshanensis*	OR786306	1.83/1.88G	147,794	85,026	11,032	25,867	110	30	4	OR073412	655
*G.lihengiae*	OR002168	1.60/1.59G	147,940	84,863	11,165	25,956	101	29	4	OR073408	656
*G.lihengiae*	OR002169	2.01/2.00G	147,934	84,829	11,173	25,966	101	29	4		655
*G.nanchuanensis*	OR002176	1.56/1.46G	148,001	84,942	11,045	26,007	110	30	4	OR073410	89
*G.* sp.	OR002172	1.69/1.73G	147,706	84,938	11,032	25,867	110	30	4		621
*G.* sp.	OR002174	1.70/1.80G	147,708	84,938	11,032	25,869	101	29	4	OR073409	654

### ﻿Phylogenetic relationships within *Neottia*

**Figure 1.**
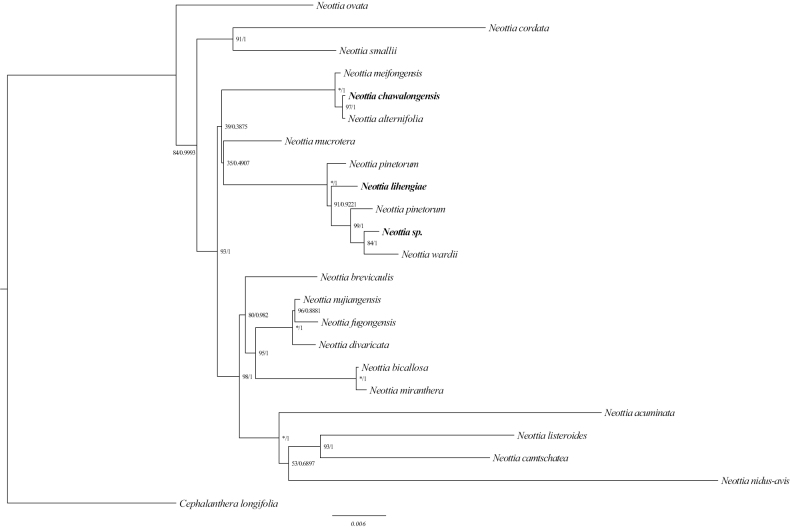
Phylogenetic relationships of *Neottia* species based on the nrITS, *mat*K and *rbc*L. The ML and BI trees have the same topology. Numbers at nodes are Bayesian posterior probabilities and bootstrap percentages, respectively. “*” represents 100% support with newly sequenced species are shown in bold italics.

Phylogenetic relationships based on combined nrITS and plastid DNA (*mat*K, *rbc*L) data indicated that *Neottia* s.l. is monophyletic with moderate support (BP = 84, PP = 0.9993). Within the sampled species, the widespread and temperate *N.ovata* diverged initially, which is consistent with the previous study by Zhou and Jin (2018), followed by the clade of *N.cordata* (L.) Rich. and *N.smallii* (Wiegand) Szlach. The newly discovered species *N.chawalongensis* (Fig. 1) is sister to *N.alternifolia* (King & Pantl.) Szlach. (PP = 1, BP = 97). Together, they constitute the sister clade of *N.meifongensis* (H.J.Su & C.Y.Hu) T. C. Hsu & S. W. Chung. The second new species, *N.lihengiae* (Fig. 1) is clustered with *N.pinetorum* (Lindl.) Szlach. the unidentified *Neottia* sp. and *N.wardii* (Rolfe) Szlach. (PP = 0.92, BP = 91).

### ﻿Phylogenetic relationships of *Papilionanthe*

In the overall matrix of *Papilionanthe*, 75 sequences were obtained (13 nrITS sequences and 14 *mat*K, 14 *trn*L-*trn*F, 11 *psb*A-*trn*H, 11 *atp*I-*atp*H, 9 *trn*S-*trnf*M, and 3 *rbc*L sequences, respectively), and the combined dataset of 7 markers comprised 8558 aligned nucleotides, 790 bp from nrITS and 7768 bp from plastid regions, respectively.

**Figure 2.**
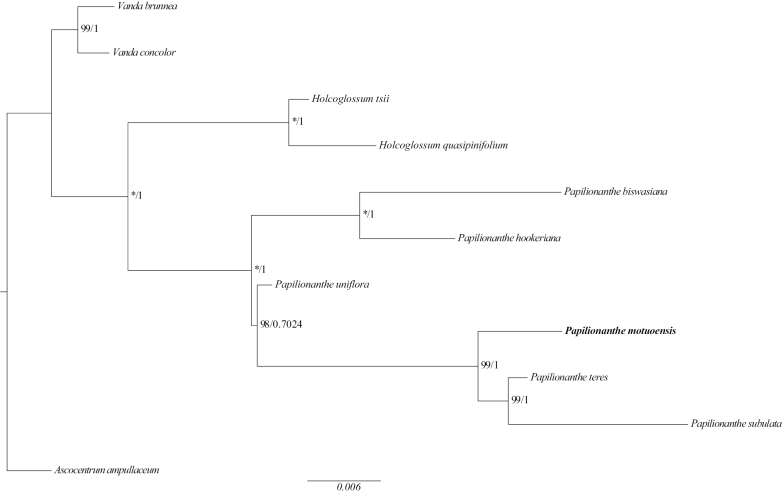
Phylogram of the genus *Papilionanthe* based on ML and BI analyses of the combined nrITS and plastid *mat*K, *trn*L-*trn*F, *psb*A-*trn*H, *atp*I-*atp*H, *trn*S-*trnf*M, *rbc*L sequences. The ML and BI trees are identical and the BP and PP values are given beside the nodes. “*” indicates 100% bootstrap support.

The concatenated tree of nrITS and its plastid data show that *Papilionanthe* is monophyletic. The main clade of *Papilionanthe* is divided into two subclades (Fig. 2). In the first, *P.biswasiana* (Ghose & Mukerjee) Garay and *P.hookeriana* (Rchb.f.) Schltr. are sister species. In the second subclade, *P.uniflora* (Lindl.) Garay diverged first while the newly sequenced *P.teres* is well supported as sister to *P.subulata* (Willd.) Garay (BP = 99, PP = 1). Collectively it is sister to the new species *P.motuoensis* (see below) (BP = 99, PP = 1). The BI and maximum likelihood (ML) trees yield the same topology. The posterior probabilities and bootstrap probabilities values are high, indicating a high degree of confidence for the result. However, this topology is inconsistent with the previous study by Zhang et al. (2013). The difference between these topologies was the position of *P.hookeriana* and *P.biswasiana*, now consisting of a sister group in this study.

### ﻿Phylogenetic relationships of *Gastrochilus*

**Figure 3.**
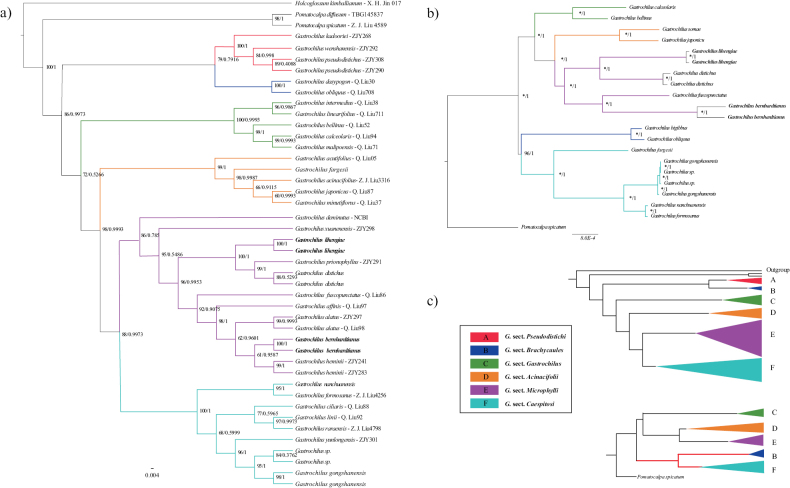
**a** Phylogenetic relationships in genus *Gastrochilus* based on nrDNA ITS and plastid *mat*K, *trn*L-*trn*F, *psb*A-*trn*H, *psb*M-*trn*D sequences. ML and BI trees have the same topology and BP and PP are given beside the branches **b** phylogenetic relationships of *Gastrochilus* based on the complete plastomic sequences. All nodes are supported with a posterior probability (pp) of 1.0. “*” indicates 100% bootstrap support **c** phylogenetic tree based on combined nrITS and plastid DNA markers and conflicting topologies (clade B and clade F) are highlighted.

A total of 41 *Gastrochilus* species were included in this study to represent all six sections, 12 accessions representing nine species were newly generated in this study. Their relationships were confirmed using a combined dataset of nrITS and plastid *mat*K, *trn*L-*trn*F, *psb*A-*trn*H, *psb*M-*trn*D sequences (Fig. 3a). The detailed sequence information is listed in Suppl. material 4, and the best-fit model selected by jModeltest is given in Table 2. Both RAxML and Bayesian inference (BI) analyses of the concatenated sequence supermatrix produced similar topologies for the *Gastrochilus* species. *Gastrochilus* s.l. is strongly supported as monophyletic with high posterior probabilities (PP) and bootstrap probabilities (BP) (Fig. 3a). The genus was subdivided into six well-supported clades, the earliest diverging clade is clade A (G.sect.Pseudodistichi), successively followed by clade B (G.sect.Brachycaules) (BP = 79, PP = 0.7916), clade C (G.sect.Gastrochilus) (BP = 100, PP = 0.9993) and clade D (G.sect.Acinacifolii) (BP = 99, PP = 1). Clades E and F are sister to each other and they together comprise a clade sisiter to clade D. Our two new species, *G.lihengiae* (see below) and *G.bernhardtianus* (see below) are resolved as distinct species in clade E (G.sect.Microphylli) in all data sets. *Gastrochilusdistichus* (Lindl.) Kuntze + *G.prionophyllus* H. Jiang, D. P. Ye & Q. Liu is sister to *G.lihengiae* while *G.heminii* M. Liao, B. Xu & Yue.H. Cheng is sister to *G.bernhardtianus* and they consisting a sister group to *G.alatus*. Within clade F, two samples of *G.gongshanensis* Z.H.Tsi and two unidentified species from the Dali, Yunnan form a distinct subclade, which is sister to *G.yunlongensis* W. H. Rao, L. J. Chen & Z. J. Liu.

**Table 2.** Best-fit models and parameters for each genus.

Genus	Region	AIC select model	Base frequencies				Base frequencies						p-inv (I)	Gamma shape (G)
			A	C	G	T	A-C	A-G	A-T	C-G	C-T	G-T		
*Neottia*	ITS, *mat*K, *rbc*L	GTR+I+G	0.2907	0.1881	0.1987	0.3226	1.1924	1.7728	0.3011	0.2107	2.0924	1.0000	0.4320	0.9250
*Papilionanthe*	ITS, *mat*K, *trn*L-F, *psb*A-*trn*H, *trn*S-*trnf*M	GTR+G	0.3083	0.1866	0.1675	0.3376	1.1439	1.3935	0.4909	0.4617	1.3426	1.0000		0.3190
*Gastrochilus*	Plastome	GTR+I+G	0.3129	0.1844	0.1785	0.3242	1.0779	1.2742	0.3062	0.2105	1.1734	1.0000	0.7880	0.8410
*Gastrochilus*	ITS, *mat*K, *trn*L-F, *psb*A-*trn*H, *psb*M-*trn*D	GTR+I+G	0.3207	0.1879	0.18	0.3114	0.8602	1.6575	0.3075	0.5360	1.8524	1.0000	0.1610	0.0210

In the present study, analysis of 20 complete chloroplast genomes of *Gastrochilus* specimens provide a wealth of information to determine phylogenetic relationships within this genus, including a fully resolved phylogenetic tree with almost 100% bootstrap values and 1.00 posterior probabilities, and are better supported than in the studies of Liu et al. (2019) and Zhang et al. (2023) based on nrITS and plastid sequences (Fig. 3b). However, these phylogenetic relationships based on whole plastomes and chloroplast sequences suggest different topologies, particularly among the relationship between Clade B and subclade including *G.fargesii* (Fig. 3c). The analysis by Zhang et al. (2023) suggested that *G.obliquus* diverged early in the clade B with *G.formosanus* falling into clade F (Fig. 3a). In contrast, our plastome-based topology showed that species of clade B recovered as sister to clade F with high statistic support (Fig. 3c). One explanation is the difference in information sites, and the other possibility is sampling size. More samples with plastomic data should be used in future study to resolve the difference.

### ﻿Conservation status

We preliminarly assesed the conservation status of the five new species using the IUCN Red List Categories and Criteria (IUCN 2022). *Neottialihengiae* is known from two sites with a population of >2,000 individuals in an area of ca. 80 square kilometers scattered under a protected, evergreen broadleaved forest and a mossy dwarf forest in northwestern Yunnan, respectively. Based on population size and healthy habitats, the conservation status is proposed as Least Concern (LC). In contrast, the remaining four species, are known only from type localities and adjacent areas. For each of these species, only one or two populations with few individuals were detected during our two to three field surveys. More extensive fieldwork is needed to objectively assess their conservation status.. Therefore, the status of all remaining species are temporarily rated as Data Deficient (DD).

### ﻿Taxonomic treatments

#### ﻿*Neottia* Guett.

##### ﻿1. Neottia lihengiae

Taxon classificationPlantaeAsparagalesOrchidaceae

J.D.Ya, H.Jiang & D.Z.Li sp. nov.

**Figure 4.**
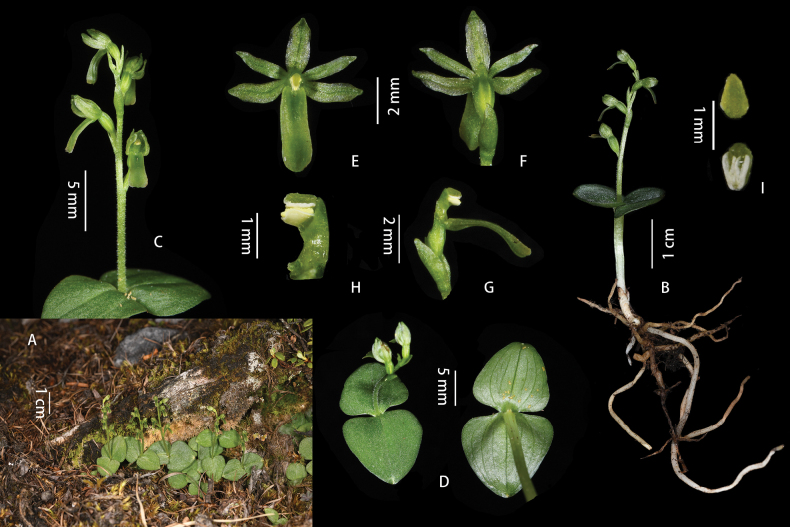
*Neottialihengiae* J.D.Ya, H.Jiang & D.Z.Li, sp. nov. **A** habitat **B** plant **C** inflorescence **D** leaves **E** flower (front view) **F** flower (dorsal view) **G** column and labellum **H** column **I** anther cap. Photographed by J.-D. Ya.

9E91EFA0-BE0D-5065-8D5D-2192327335FC

urn:lsid:ipni.org:names:77331171-1

(李恒对叶兰 Li Heng Dui Ye Lan) Fig. 4

###### Diagnosis.

*Neottialihengiae* is morphologically similar to *N.biflora* (Schltr.) Szlach., but can be distinguished by its smaller plant size, ca. 5.5–9.0 cm tall (vs. 10–13 cm tall), its lax rachis of 2–5–flowered (vs. 1- or 2-flowered), floral bracts and sepals longer than their pedicel (vs. shorter than pedicel), smaller flowers with sepals and petals connivant and ca. 3.0 mm long (vs. spreading and ca. 6.0–7.0 mm long). The outer surfaces of the sepals are not carinate (vs. carinate). The labeullum is ligulate and its midvein is not thickened (vs. cuneate and midvein slightly thickened). The rostellum is almost equal to the anther (vs. distinctly shorter than the anther).

###### Type.

China. Yunnan Province, Diqing Prefecture, Shangri-La County, Tianbao mountain, 3800 m, under shrubs of a scree slope, 4 July 2020, J.-D. Ya et al. 20CS19095 (Holotype: KUN! isotype: KUN!)

###### Description.

Terrestrial, autotrophic herbs, 5.5–9.0 cm tall. Rhizome with many elongate, filiform roots. Stem erect, slender, usually with 1 or 2 membranous ca. 8.0 mm long tubular sheaths at its base. Leaves 2, opposite, borne above the middle of the plant, 7 veined from the base, subsessile, broadly ovate or broadly ovate-triangular, unequal in size, the larger leaf ca. 1.2 × 1.2 cm, the smaller one ca. 1.0 × 1.0 cm, with bases rounded and apices acute. Peduncle 0.7–1.2 cm, puberulous, rachis 1.2–1.8 cm, laxly 2–5-flowered; floral bracts ovate-lanceolate, concave, longer than the pedicel, 3–4 × ca. 0.8 mm, apex acute to acuminate. Flowers resupinate, uniformly green; pedicel and ovary 2.0–3.0 mm long, glabrous; sepals and petals connivent. Dorsal sepal ovate-lanceolate, ca. 3.2 × 1.1 mm, 1-veined, apex subacute; lateral sepals lanceolate, slightly oblique, ca. 3.5 × 0.8 mm, 1-veined, apex acute. Lateral petals linear-lanceolate, ca. 3.0 × 0.6 mm, 1-veined, apices subacute; labellum ligulate, ca. 4.0 × 1.6 mm, entire to shallowly notched or emarginate at apex, usually with a minute tooth in the notch. Column slightly arcuate, ca. 1.7 mm long, anther inclined toward rostellum, ca. 0.9 mm; rostellum spreading forward, nearly as long as the anther.

###### Phenology.

Flowers from June to July.

###### Etymology.

Named in honor of late Prof. Li Heng, a Chinese botanist who made significant contributions to our understanding of plant diversity and phytogeography of the Gaoligong Mountains at the border between China and Myanmar (Guo et al. 2023).

###### Distribution and habitat.

It is known from Northwest Yunnan including Lijiang and Diqing. It grows under shrubs colonizing scree slopes at elevations of 3700–3800 m.

###### Additional specimen examined.

China. Yunnan Province, Lijiang City, Gucheng District, Dadong Xiang, 3192 m, in the scree slope area under the forest dominated by *Pinusdensata* Mast. 17 June 2017, H. Jiang and W.P. Zhang 08835 (paratypes: YAF!); Yunnan Province, Diqing Prefecture, Shangri-La County, Tianbao mountain, 3719 m, under the shrub of scree slope, 15 Aug. 2018, C. Liu et al. 18CS17401 (paratypes: KUN!). *N.biflora*: China. Sichuan, Dongrergo, K. A. H. Smith 3656 (isotypes, PE00027184!). *N.tianschanica*: China. Xinjiang Uygur Autonomous Region, Tian-Shan, 18 July 1957, K.-Z. Guan 172 (holotype, LE 01012234!); China. Xinjiang Uygur Autonomous Region, Urumqi, Houxia Zhen, 2161 m, J.D. Ya et al. 17CS16209 (KUN1437961!).

##### ﻿2. Neottia chawalongensis

Taxon classificationPlantaeAsparagalesOrchidaceae

J.D.Ya & D.Z.Li sp. nov.

**Figure 5.**
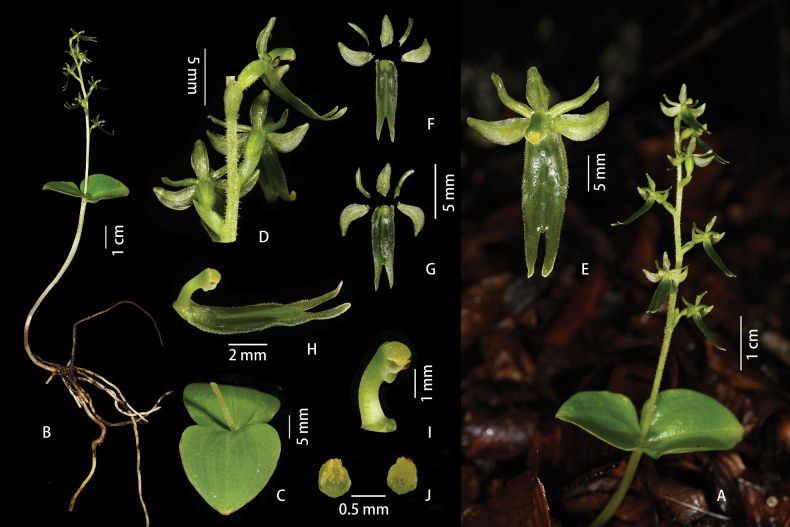
*Neottiachawalongensis* J.D.Ya & D.Z.Li, sp. nov. **A, B** plant **C** leaves **D** inflorescence **E** flower (front view) **F** adaxial sepals, petals and lip **G** abaxial sepals, petals and lip **H** column and lip **I** column **J** anther cap. Photographed by J.-D. Ya.

FECD1EEB-3A25-50F9-8986-C156B026525D

urn:lsid:ipni.org:names:77331172-1

(察瓦龙对叶兰 Cha Wa Long Dui Ye Lan) Fig. 5

###### Diagnosis.

*Neottiachawalongensis* is similar to *N.pinetorum* (Lindl.) Szlach., but differs in having floral bracts longer than its pedicel (vs. shorter or as long as pedicel), a reduced pedicel ca. 1.9 mm (vs. 4–6 mm), and a shorter but pubescent ovary, ca. 2.8 mm (vs. glabrous, 3–4.5 mm). The labellum is lanceolate (vs. obovate-cuneate, oblong-cuneate, sublinear-cuneate, or oblanceolate), densely papillate (vs. slightly papillate), with labellum lobes narrowly lanceolate and apices acuminate (oblong-ovate and apices obtuse-rounded) while its sinus usually lacks a short tooth between the lobes.

###### Type.

China. Tibetan Autonomous Region, Linzhi City, Chayu County, Chawalong Township, 3757 m, under the shrub of scree slope, 21 July 2022, J.-D. Ya et al. 22CS22851 (KUN!)

###### Description.

Terrestrial, autotrophic plants, 9.0–13.5 cm tall. Rhizome with many elongate, filiform roots. Stem 5.0–7.0 cm, erect, slender, ridged, usually with 1 or 2 membranous, long, tubular sheaths at its base. Leaves 2, opposite, borne in the middle of the plant, 5 veined from the base, subsessile, broadly ovate or broadly ovate-triangular, ca. 2.0 × 1.8 cm in diameter. Peduncle 1.8–2.4 cm, puberulous, rachis 2.7–4.3 cm, held laxly bearing 7–9-flowered; floral bracts ovate, concave, longer than the pedicel, 3–4 mm long with acute apices. Flowers resupinate, uniformly green; pedicel ca. 1.9 mm, glabrous to sparsely pubescent; ovary ca. 2.8 mm, pubescent with sepals and petals widely spreading. Dorsal sepal narrowly elliptical, ca. 3.5 × 1.3 mm, 1-veined, apex obtuse; lateral sepals narrowly elliptic-falcate, ca. 3.5 × 1.5 mm, with an obtuse apex. Lateral petals linear, ca. 3.0 × 0.4 mm, apices subacute; labellum pendulous, lanceolate, ca. 8.0 × 2.2 mm, margins densely papillate, apex deeply 2-lobed with lobes parallel, narrowly lanceolate, ca. 2.6 × 0.7 mm, and apices acuminate; disk with a thickened longitudinal channel extending from the base of the labellum almost to the sinus. Column slightly arcuate above the middle, 3.5 mm long; anther inclined towards the rostellum, ca. 0.8 mm; rostellum spreading forward.

###### Phenology.

Flowers from July to August.

###### Etymology.

The specific epithet “chawalongensis” refers to the type locality Chawalong (Cawarong) Township.

###### Distribution and habitat.

At present, this new species is only found in Chawalong, Chayu, Tibet (Xizang), China. It is a predominantly terrestrial species growing on the scree slopes under the forest of *Abies* and *Picea* at an elevation of 3757 m a.s.l. It appears to be locally abundant with other orchid species including *Ponerorchischusua* (D. Don) Soó, *Galearisspathulate* (Lindl.) P. F. Hunt, *Cypripediumwardii* Rolfe, *C.bardolphianum* W.W.Sm. & Farrer and *C.flavum* P. F. Hunt & Summerh.

###### Additional specimen examined.

*N.pinetorum*: India. Sikkim, 10–11000 feet., J. D. Hooker 355 (holotype, K000974204!, isotype, AMES 00101020!); China. Yunnan, upper Kiukiang valley, 2500 m, T.T.Yu 19644 (PE00027188!). *N.bambusetorum*: China, Yunnan, Prope fines Tibeto-Birmanicas inter fluvios Lu-djiang (Salween) et Djiou-djiang (Irrawadi or. sup.), in jugi Tschiangschel, 27°52', lateris orientalis regione (frigide) temperata in bambusetis, 3275–3350 m, Hand.-Mazz.9238 (holorypus, WU0061594!)

#### ﻿*Papilionanthe* Schltr.

##### ﻿3. Papilionanthe motuoensis

Taxon classificationPlantaeAsparagalesOrchidaceae

J.D.Ya & D.Z.Li sp. nov.

**Figure 6.**
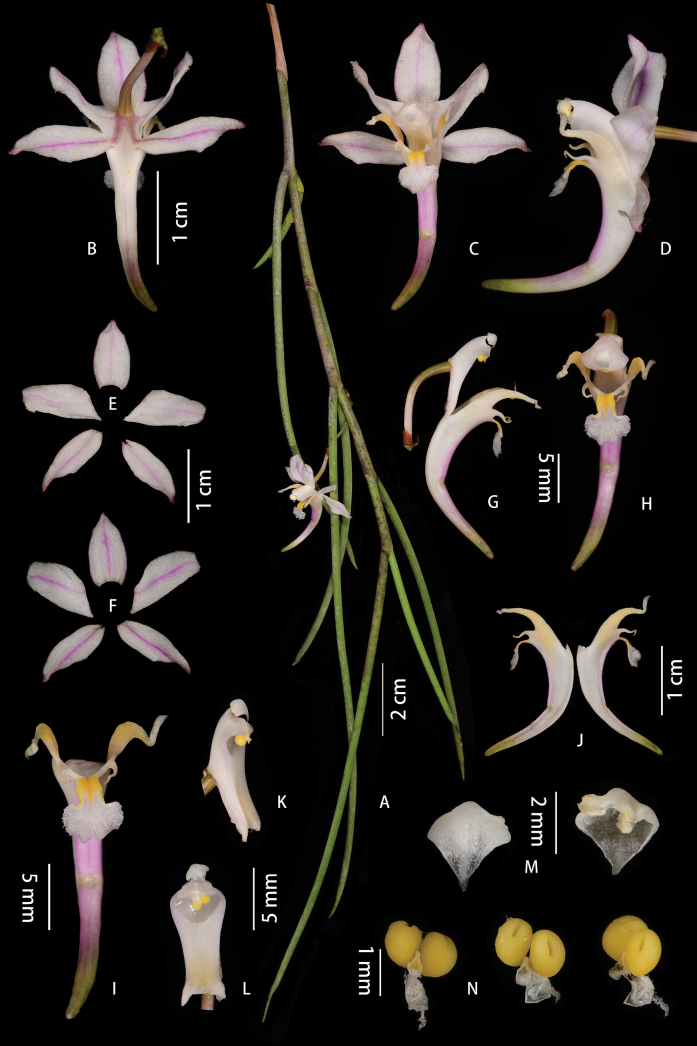
*Papilionanthemotuoensis* J.D.Ya & D.Z.Li, sp. nov. **A** plant **B** flower (dorsal view) **C** flower (front view) **D** flower (lateral view) **E** abaxial sepals and petals **F** adaxial sepals and petals **G** column and lip (lateral view) **H** column and lip (front view) **I** lip (front view) **J** lip (rip cutting) **K** column (lateral view) **L** column (front view) **M** anther cap **N** pollinarium. Photographed by J.-D. Ya.

**Figure 7.**
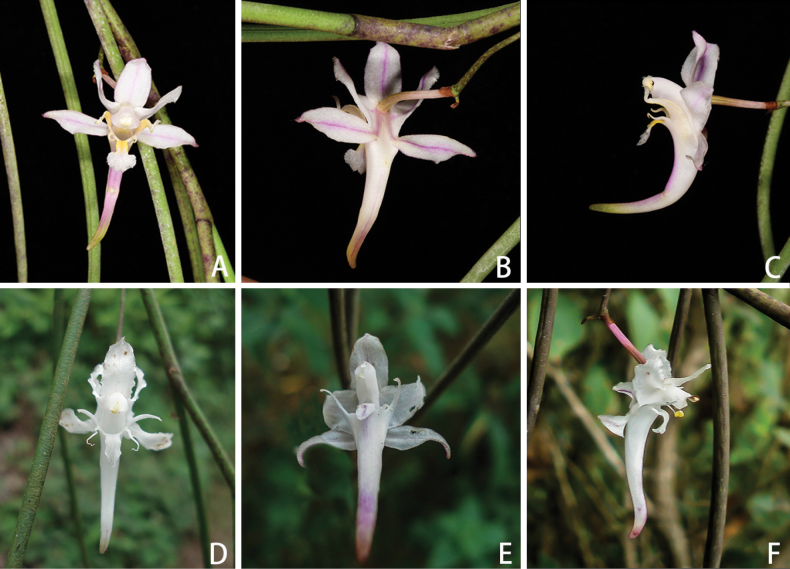
**A–C***Papilionanthemotuoensis***D–F***Papilionantheuniflora* (Nepal). Photographed by: **A–C** J.-D. Ya **D–F** Bhakta Bahadur Raskoti.

5F97E7EB-BF0A-5454-835B-65DEA37917A4

urn:lsid:ipni.org:names:77331173-1

(墨脱凤蝶兰 Mo Tuo Feng Die Lan) Figs 6 , 7A–C

###### Diagnosis.

*Papilionanthemotuoensis* is similar to *P.uniflora* (Lindl.) Garay but differs in having a glabrous pedicel and ovary (vs. glandular-pubescent). Its lateral petals are oblong-ovate (vs. oblong) with irregularly denticulate margins (vs. with undulating margins), truncate apices (vs. obtuse apices). Its labellum is white tinged with yellow (vs. uniformly white), with a subflabellate mid-lobe and a labellum base with an apically dilate to reniform claw, its apex is emarginated with an irregularly denticulate margin (vs. mid-lobe simple, oblong, apex widely cuneate).

###### Type.

China. Yunnan, Kunming, voucher from cultivated plants at Kunming Institute of Botany, CAS, 20 Oct. 2020 (flowering), J.-D. Ya BC201015 (holotype: KUN!), plants originally collected from Tibet (Xizang), Linzhi City, Motuo County, 1625 m, at the edge of a subtropical, evergreen, broadleaved forest.

###### Description.

Stems pendulous, terete, to 50 cm, 2.0 mm in diam. branched, enclosed in leaf sheaths. Leaves laxly alternate, terete, 9–16 × 0.2 cm, base with amplexicaul-sheathing, apex apiculate; sheaths tubular, 2.0–2.8 cm long, glabrous. Inflorescence ca. 1.5 cm, usually 1–2-flowered; peduncle slender, ca. 1.3 cm; floral bracts ovate-triangular, 1.5 × 1.2 mm. Flowers 2.5 cm in diam. sepals and lateral petals white, mid-vein pink, labellum white tinged with yellow, its spur with a whitish and/or pink tinge, apex yellowish green. Pedicel and ovary, ca. 1.2 cm long, glabrous. Dorsal sepal ovate, ca. 1.0 × 0.5 cm, acute, 5-veined; lateral sepals oblong, slightly falcate, ca. 1.1 × 0.4 cm, acuminate, 5-veined; lateral petals oblong-ovate, 1.1 × 0.6 cm, margin irregularly denticulate, apices truncate, 7-veined; labellum adnate to column foot, 3-lobed; lateral lobes deeply bifid, unequal, linear, acute, long lobule ca. 7.0 × 2.0 mm, short lobule ca. 4.0 × 1.0 mm; mid-lobe spreading, subflabellate, ca. 4.8 × 5.0 cm, base with a claw ca. 2.2 × 2.0 mm, apical dilate to reniform, apex emarginate, margin irregularly denticulate; spur slightly curved forward, cylindrical, ca. 22.0 × 3.5 mm, narrowing towards the terminus, its interior pubescent. Column 7.0 × 3.0 mm, foot ca 5.2 mm, with narrowly and entire wings decurrent to foot; anther cap galeate with a acuminate apex, 2.0 × 2.5 mm; pollinia 2, subglobose, ca. 1.0 mm in diameter, waxy, porate, attached by a stipe to a broad cellular viscidium.

###### Phenology.

Observed flowering in October.

###### Etymology.

The specific epithet “*motuoensis*” refers to the type locality Motuo (Medog) County.

###### Additional specimens examined.

Tibet (Xizang), Linzhi City, Motuo County, Bangxin Xiang, 1330 m, from subtropical, evergreen, broadleaved forest. Oct. 2019, M.-K. Li and W. Wang 2019343 (paratypes, Herbarium of Tibet Agricultural and Animal Husbandry University, No. 8 Xueyuan Road, Bayi District, Nyingchi, Tibet). *P.uniflora*: Nepal. Gosain Than, N. Wallich no. 1993 (K001114863!); India, Mao, C.B. Clarke 41790 (K000891405!)

###### Distribution and habitat.:

 The new epiphytic species was found only in Motuo County, Tibet (Xizang), China, growing on limbs in a subtropical, evergreen, broadleaved forest at elevations of 1300–1650 m.

#### ﻿*Gastrochilus* D.Don

##### ﻿4. Gastrochilus lihengiae

Taxon classificationPlantaeAsparagalesOrchidaceae

J.D.Ya, Ting Zhang & Z.D.Han sp. nov.

**Figure 8.**
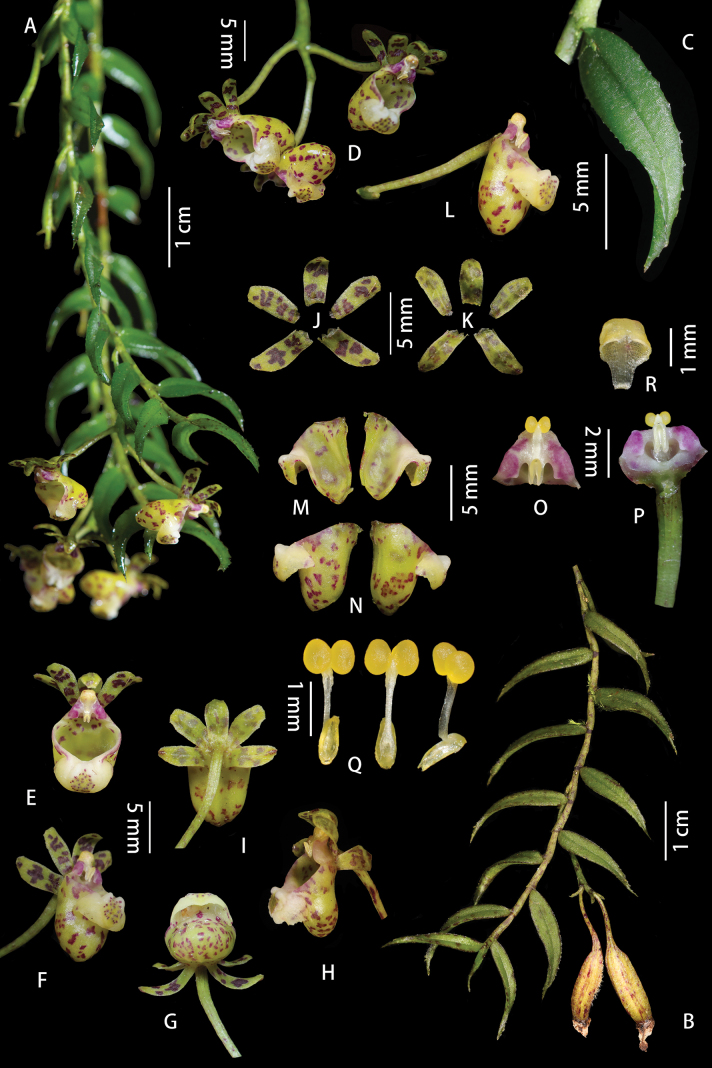
*Gastrochiluslihengiae* J.D.Ya, Ting Zhang & Z.D.Han, sp. nov. **A** flowering plant **B** fruiting plant; Stem **C** leaf **D** inflorescence **E–I** flower (different view) **J** adaxial sepals and petals **K** abaxial sepals and petals **L** column and lip **M, N** lip (rip cutting) **O, P** column **Q** pollinarium **R** anther cap. Photographed by J.-D. Ya & Z.-D. Han.

**Figure 9.**
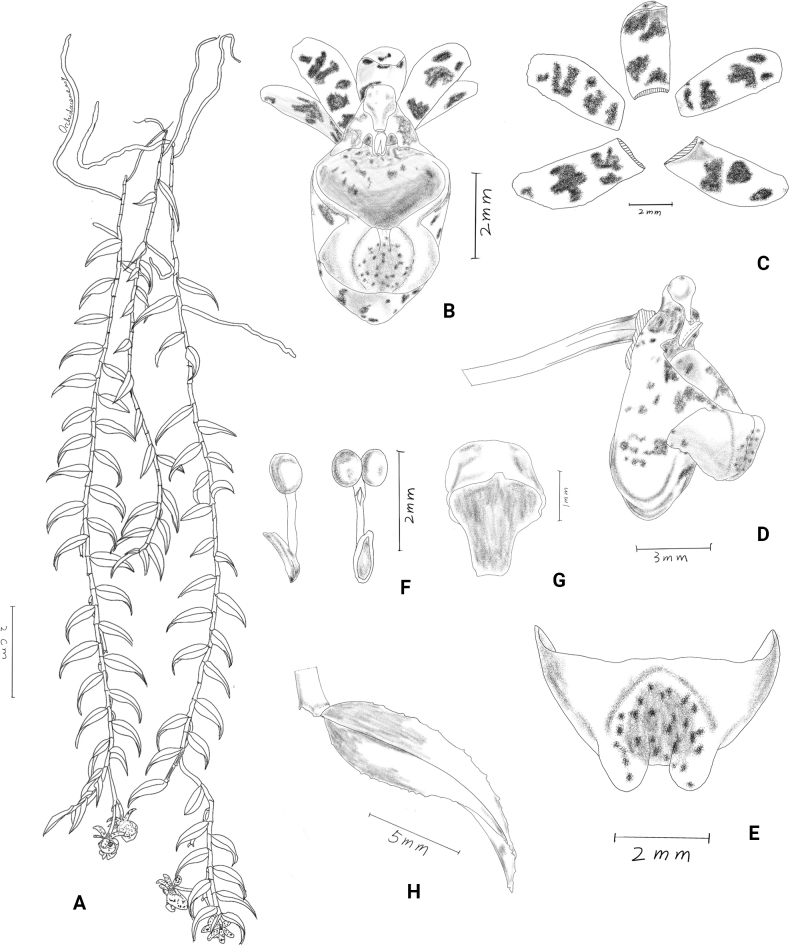
*Gastrochiluslihengiae* J.D.Ya, Ting Zhang & Z.D.Han, sp. nov. **A** plant **B** flower **C** sepals and petals **D** column and lip **E** epichile **F** pollinarium **G** anther cap **H** leaf. Drawn by Z.-D. Han.

A3D99443-4319-5347-91CF-EE5EC5E27F20

urn:lsid:ipni.org:names:77331174-1

(纤细盆距兰 Xian Xi Pen Ju Lan) Figs 8 , 9

###### Diagnosis.

The floral morphology of *Gastrochiluslihengiae* is similar to *G.distichus* (Lindl.) O. Kuntze and *G.prionophyllus* H. Jiang, D. P.Ye & Q. Liu, but can be distinguished from the former by its narrower leaves, blades 0.25–0.35 cm wide (vs. 0.4–0.6 cm), and distinctly serrate leaf margins (vs. entire), with acuminate and mucronate apices (vs. apex acute bearing 2 or 3 awns). The lateral petals are narrowly oblong (vs. subobovate). The labellum with a hypochile, ca 7.0 mm (vs. 4.0 mm). The outside of the hypochile with three ridges (vs. glabrous), and from the latter by its falcate-lanceolate (vs. ovate) leaves with mucronate apices (vs. apex with 2 unequally awns), the lateral petals are narrowly oblong (vs. subobovate), the central cushion on the epichile of the labellum is not thickened (vs. thickened), while the outer surface of its hypochile has three ridges (vs. glabrous).

###### Type.

China. Yunnan Province, Nujiang Prefecture, Gongshan County, Cikai Township, 1935 m, in the montane moist evergreen broad-leaved forest, 24 Apr. 2020, J.-D. Ya et al. 22CS21828 (Holotype: KUN! isotype: KUN!)

###### Description.

Epiphytic herbs, stem pendulous, to 20 cm long, ca. 1.0–1.5 mm in diameter, slender, with 0.5–0.6 cm internodes, often branched with tiny red-purple spots. Leaves alternate, distichous, falcate-lanceolate, ca. 1.6–1.8 × 0.25–0.35 cm, the margin significantly serrate with an acuminate and mucronate apex. Inflorescences several, held opposite to nearly opposite the leaves, subumbellate, 1–3-flowered; peduncle 0.7–1.0 cm, slender, upper part enlarged, lower part with 2 cupular sheaths; floral bracts ovate, ca. 1.0 mm; pedicel and ovary 1.0–1.1 cm. Flowers yellow-green, with reddish brown spots. Dorsal sepal concave, oblong-ovate, ca. 4.0 × 2.0 mm, apex obtuse; lateral sepals concave, narrowly oblong, ca. 5.5 × 1.8 mm, apex obtuse; lateral petals narrowly oblong, 4.0 × 1.8 mm, apices subtruncate. Labellum subdivided into an epichile and a saccate hypochile; the epichile subovate triangular, ca. 5.0 × 2.5 mm, adaxially glabrous, with a central cushion and 2 conic calli near its base, the margin entire to irregularly denticulate, apex rounded; the hypochile subcupular, ca. 7.0 mm tall and ca. 5.2 mm in diam. outside with three ridges from the base of the column to its apex. Column stout, ca. 2.0 mm long, with rounded-auriculate wings at the base; anther cap narrowed into a beak towards its apex; the rostellum bilobed with an acuminate tip, and a horn-like awn arising from the center of each lobe; pollinarium ca 2.1 mm long; pollinia 2, yellow, 0.8 × 0.5 mm, almost hemispheric with a depression at the center; stipe elongate, obovate, ca.1.5 mm long; cellular viscidium elliptic, 1.0 × 0.5 mm. Capsules cylindrical, ca. 1.5 × 0.6 cm.

###### Phenology.

Flowering from March to April, while the fruits matured in March in the following year.

###### Etymology.

Named in honor of late Prof. Li Heng for her contributions to the orchid flora of Yunnan (Guo et al. 2023).

###### Distribution and habitat.

At present, two populations of this new species were found in Gongshan County, Yunnan, China. It is epiphytic on tree trunks in the mixed evergreen broad-leaved forest or montane moist evergreen broad-leaved forest at an elevation of 1900–2100 m.

###### Additional specimens examined.

China. Yunnan Province, Nujiang Prefecture, Gongshan County, Dulongjiang Xiang, 2051 m, in the mixed evergreen broad-leaved forest, 4 Mar. 2023, Ting Zhang et al. 23CS24145 (paratypes, KUN!). *G.distichus*: India. Skimm, J.D. Hooker 206. (holotype: K000873754!). *G.prionophyllus*: China. Yunnan, Malipo County, Xia jinchang town, limestone forest, 1550–1650 m a.s.l., epiphytic on tree trunks or on rocks, 15 Mar. 2016, Qiang Liu 359 (holotype, HITBC!). *G.fargesii*: China. Sichuan, Tschen-keou-tin, P.G. Farges 1236 (type, K00083803! isotype, AMES00271835!).

##### ﻿5. Gastrochilus bernhardtianus

Taxon classificationPlantaeAsparagalesOrchidaceae

J.D.Ya & D.Z.Li sp. nov.

**Figure 10.**
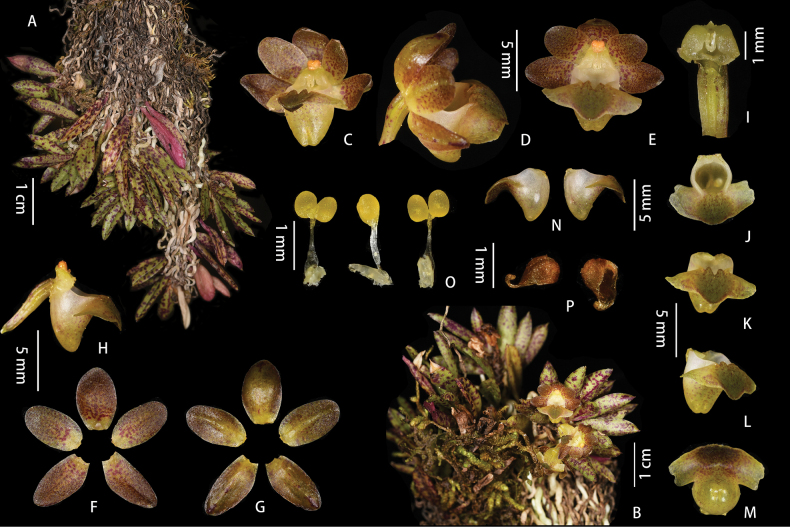
*Gastrochilusbernhardtianus* J.D.Ya & D.Z.Li, sp. nov. **A** plant **B** inflorescence **C–E** flower **F** adaxial sepals and petals **G** abaxial sepals and petals **H** column and lip **I** column **J–M** lip **N** lip (rip cutting) **O** pollinarium **P** anther cap. Photographed by J.-D. Ya.

**Figure 11.**
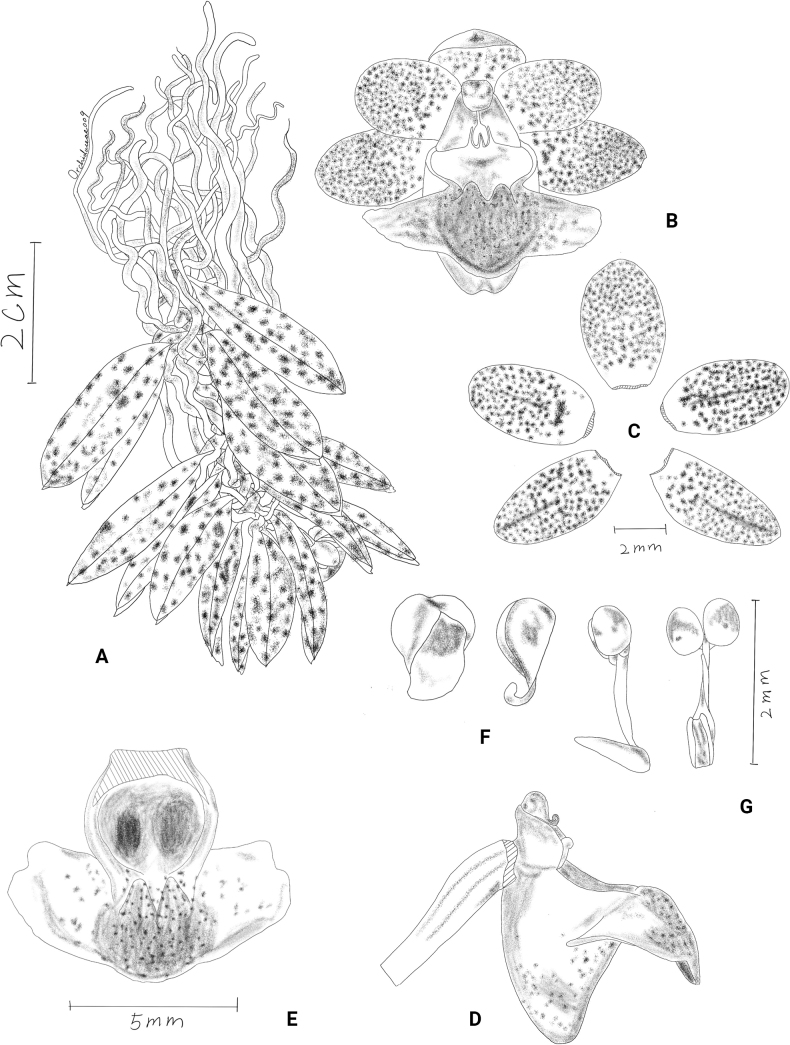
*Gastrochilusbernhardtianus* J.D.Ya & D.Z.Li, sp. nov. **A** plant **B** flower **C** sepals and petals **D** column and lip **E** lip **F** anther cap **G** pollinarium. Drawn by Z.-D. Han.

**Figure 12.**
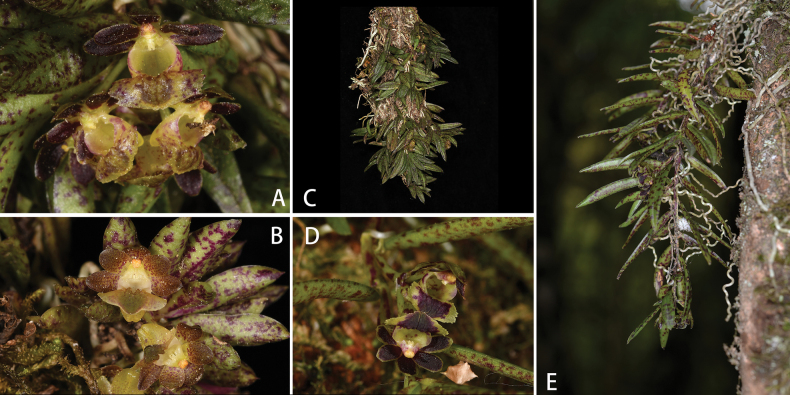
Comparison of two taxa of *Gastrochilus***A–C***G.bernhardtianus* with various colours in different areas **D, E***G.affinis*. Photographed by J.-D. Ya.

E448DD77-6942-5DE4-8D11-1A71F788B72A

urn:lsid:ipni.org:names:77331175-1

(丽江盆距兰 Li Jiang Pen Ju Lan) Figs 10 , 11 , 12 A–C

###### Diagnosis.

*Gastrochilusbernhardtianus* is similar to *G.affinis* (King & Pantl.) Schltr. in floral morphology, but can be distinguished by its shorter peduncle, ca. 0.3 cm (vs. 1.5–2.0 cm), pedicel and ovary ca. 4.5 mm (vs.0.6–1.3 cm). Sepals and lateral petals dark yellowish-green with densely purplish-red marks or spots flushed brown to purplish brown (vs. green flushed with brown to purplish brown). The dorsal sepal elliptic, ca 3.4 mm wide (vs. elliptic-oblong, 1.0–1.3 cm wide), lateral sepals narrowly ovate, ca. 5.5 × 2.8 mm (vs. elliptic-ovate, 3.5–4.0 × 0.7–1.3 mm). Lateral petals narrowly oblong, ca. 5.2 × 2.7 mm (vs. ovate-elliptic to elliptic, 3.0–4.0 × 1.0–1.3 cm). Labellum with purplish-red spots (vs. yellowish to greenish-yellow marks) and yellowish-green calli (vs. brown to purplish brown) with a transversely oblong epichile (vs. broadly subtriangular) and a green center (vs. deep purple to purplish-brown).

###### Type.

China. Yunnan Province: Lijiang Prefecture, Yulong County, Yunshanping, 3308 m, in cold-temperate, evergreen conifer forest, 20 May 2020, J.-D. Ya et al. 20CS19022 (Holotype: KUN!)

###### Description.

Epiphytic herb, stem pendulous, with purplish spots, ca. 5.0 cm long, 1.5–2.0 mm in diameter. Leaves distichous, blade oblong-lanceolate, with purple-red spots on the abaxial leaf surface, 1.8–2.5 × 0.4–0.7 cm, base sheathing, apex acute and slightly trilobate. Racemes axillary, sub-umbellate, 1–2 flowered; peduncle ca. 0.3 cm, with purple-red spots; floral bracts ovate-triangular, ca. 1.0 mm; pedicel and ovary yellow-green with purple-red spots, ca. 4.5 mm. Flower densely marked with purplish-red spots flushed with brown to purplish brown, sepals and lateral petals dark yellowish-green. Dorsal sepal elliptical, ca. 5.2 × 3.4 mm, apex obtuse; lateral sepals narrowly ovate, ca. 5.5 × 2.8 mm, apices obtuse; lateral petals narrowly oblong, ca. 5.2 × 2.7 mm, apices obtuse. Labellum epichile with a green center and yellowish green margins, transversely oblong, ca. 8.0 × 2.8 mm, adaxially glabrous, with a central cushion and 2 conic calli near its base, margins erose, apex rounded; hypochile saccate, light yellowish green, subconical, ca. 5.1 mm tall and ca. 3.8 mm in diam. dorsally compressed, slightly bent outward, subacute to obtuse and shortly bifid at apex, with one internal ridge at the bottom. Column stout, ca. 2.0 mm, with rounded-auriculate wings at the base, anther cap galeate with recurved–acuminate apex, 1.2 × 0.9 mm; rostellum bilobed with an acuminated terminus; pollinarium ca 2.0 mm long; pollinia 2, yellow, 0.6 × 0.5 mm, almost hemispheric with a depression at the centre; stipe elongate, obovate, ca.1.0 mm long; cellular viscidium elliptic, 0.8 × 0.3 mm.

###### Phenology.

Flowering from May to June.

###### Etymology.

The species is named after Peter Bernhardt, pollination biologist and orchidologist, for his contributions to pollination ecology of Chinese orchids in collaboration with botanists of China. Previously Professor of Biology at St. Louis University, USA,Peter Bernhardt was the 2022 recipient of the Peter H. Raven Scientific Outreach Award (Raven 2023). Currently he works closely with colleagues in Yunnan as a research associate of the Missouri Botanical Garden, USA and as an adjunct professor at Curtin University, Perth, Western Australia.

###### Distribution and habitat.

The new species is found only in Yulong County, Yunnan, China, and epiphytic on trees of the cold-temperate, evergreen needleleaved forest dominated by *Picealikiangensis* (Franch.) E.Pritz. and *Abiesforrestii* Coltm.-Rog. at an elevation of 3300 m a.s.l.

###### Additional specimens examined.

*G.affinis*: India. Sikkim, Lachong Valey, R. Pantling 444 (K000891609!); China. Yunnan, Fugong, Jiakedi, east slope of Gaoligongshan, epiphyticon trunk, alt., 2555 m,16 May 2005, X. H. Jin6984 (PE!); Yunnan, Tengchong, 2828 m, 31 Mar 2007, X.H. Jin 8936 (PE!). *G.alatus*: China: Yunnan, Fugong, Zhuminglin, 2758 m, 16 May 2005, H.X. Jin 6998 (Holotype, PE!).

## Supplementary Material

XML Treatment for Neottia lihengiae

XML Treatment for Neottia chawalongensis

XML Treatment for Papilionanthe motuoensis

XML Treatment for Gastrochilus lihengiae

XML Treatment for Gastrochilus bernhardtianus

## ﻿Additional information

### Author ORCIDs

Ji-Dong Ya https://orcid.org/0000-0003-3389-1412

Wan-Ting Wang https://orcid.org/0009-0007-9822-4983

Yun-Long Liu https://orcid.org/0000-0003-4650-3466

Hong Jiang https://orcid.org/0000-0001-6613-8588

Zhou-Dong Han https://orcid.org/0009-0001-9431-1349

Ting Zhang https://orcid.org/0000-0003-0939-8468

Hua Huang https://orcid.org/0000-0002-7400-5651

Jie Cai https://orcid.org/0000-0003-1627-3700

De-Zhu Li https://orcid.org/0000-0002-4990-724X

### Data availability

All relevant data are within the manuscript and its Additional files. The data that support the findings of this study are openly available in the Science Data Bank at https://www.doi.org/10.57760/sciencedb.09591 or http://resolve.pid21.cn/31253.11.sciencedb.09591.

## Supplementary materials

Supplementary material 1 Voucher and GenBank accession numbers of *Neottia* samples. Data type xlsx Explanation note List of taxa and plant materials. “XXXX” indicates plastid sequences used in analysis without Genbank accession numbers. Alignment of sequences are available under the Science data bank. https://binary.pensoft.net/file/940040This dataset is made available under the Open Database License (http://opendatacommons.org/licenses/odbl/1.0/). The Open Database License (ODbL) is a license agreement intended to allow users to freely share, modify, and use this Dataset while maintaining this same freedom for others, provided that the original source and author(s) are credited.Ji-Dong Ya, Wan-Ting Wang, Yun-Long Liu, Hong Jiang, Zhou-Dong Han, Ting Zhang, Hua Huang, Jie Cai, De-Zhu Li

Supplementary material 2 List of taxa, vouchers and GenBank accession numbers of *Papilionanthe* samples downloaded from NCBI (with newly sampled sequences added, below) Data type xlsx Explanation note List of taxa and plant materials. “XXXX” indicates plastid sequences used in analysis without Genbank accession numbers. Alignment of sequences are available under the Science data bank. https://binary.pensoft.net/file/940041This dataset is made available under the Open Database License (http://opendatacommons.org/licenses/odbl/1.0/). The Open Database License (ODbL) is a license agreement intended to allow users to freely share, modify, and use this Dataset while maintaining this same freedom for others, provided that the original source and author(s) are credited.Ji-Dong Ya, Wan-Ting Wang, Yun-Long Liu, Hong Jiang, Zhou-Dong Han, Ting Zhang, Hua Huang, Jie Cai, De-Zhu Li

Supplementary material 3 Summary of publicly available *Gastrochilus* plastomes sequences in this study Data type xlsx Explanation note List of taxa and plant materials. “XXXX” indicates plastid sequences used in analysis without Genbank accession numbers. Alignment of sequences are available under the Science data bank. https://binary.pensoft.net/file/940042This dataset is made available under the Open Database License (http://opendatacommons.org/licenses/odbl/1.0/). The Open Database License (ODbL) is a license agreement intended to allow users to freely share, modify, and use this Dataset while maintaining this same freedom for others, provided that the original source and author(s) are credited.Ji-Dong Ya, Wan-Ting Wang, Yun-Long Liu, Hong Jiang, Zhou-Dong Han, Ting Zhang, Hua Huang, Jie Cai, De-Zhu Li

Supplementary material 4 Summary of publicly available *Gastrochilus* plastid sequences in this study Data type xlsx Explanation note List of taxa and plant materials. “XXXX” indicates plastid sequences used in analysis without Genbank accession numbers. Alignment of sequences are available under the Science data bank. https://binary.pensoft.net/file/940043This dataset is made available under the Open Database License (http://opendatacommons.org/licenses/odbl/1.0/). The Open Database License (ODbL) is a license agreement intended to allow users to freely share, modify, and use this Dataset while maintaining this same freedom for others, provided that the original source and author(s) are credited.Ji-Dong Ya, Wan-Ting Wang, Yun-Long Liu, Hong Jiang, Zhou-Dong Han, Ting Zhang, Hua Huang, Jie Cai, De-Zhu Li
